# P01.37. The use of DNA barcoding for identification of medicinal plant products: an example from plants used in the Southern Texas-Mexico border region

**DOI:** 10.1186/1472-6882-12-S1-P37

**Published:** 2012-06-12

**Authors:** A Schwarzbach, H Aguilar

**Affiliations:** 1 University of Texas, Brownsville, Brownsville, USA

## Purpose

DNA barcoding generates a unique identification tag for individual species based on the sequence of a short stretch of DNA. For this study we evaluated the potential of DNA barcoding methods for identification of medicinal plants using examples from the Lower Rio Grande Valley border region in Texas. Many plants that enter the supply chain are not evaluated for correct identification and better and faster methods are needed for identification of fragmented samples.

## Methods

We sequenced several genomic regions, both nuclear and chloroplast, in order to assess variability and ability to identify highly fragmented plant material or otherwise incomplete specimens. Unidentified samples were compared with data available in Genbank and with an extensive collection of known reference materials.

## Results

We successfully used phylogenetic techniques for correct placement of the unidentified samples and potential applications for this method are discussed. We compare this method with the Genbank-BLAST search technique that is widely used for sequence matching.

## Conclusion

In conclusion, DNA barcoding can be effectively used as an identification method for medicinal plant preparations that allows fast and efficient control for distributors, provides information to customers, and, in case of a poisoning accident, might aide in determining correct treatment and countermeasures. Our studies also showed that comprehensive databases for reference materials need to be carefully assembled and curated to provide reliable information for comparisons. Currently existing databases do not fulfill this requirement leading to potential misidentifications.

